# Supplementary material for: Evidence for auto-catalytic mineral dissolution from surface-specific vibrational spectroscopy
Source: Nat Commun. 2018 Aug 20;9:3316. doi: 10.1038/s41467-018-05762-9 (PMC6102271; doi:10.1038/s41467-018-05762-9)
Supplement: Supplementary file 1 — Supplementary Information [file 41467_2018_5762_MOESM1_ESM.pdf]

Supplementary Information on

Evidence for auto-catalytic mineral dissolution from surface-specific vibrational spectroscopy

Schaefer et al.

Supplementary Note 1: Center of mass frequency shift along dissolution

As illustrated in Supplementary Fig. 1, not only the SFG intensity-change along dissolution matches with that of a ~millimolar NaCl solution compared to pure water but also its corresponding frequency shift (blue double arrows). This supports our conclusion that roughly a millimole of ionic strength is generated at the interface due to dissolution of silica.

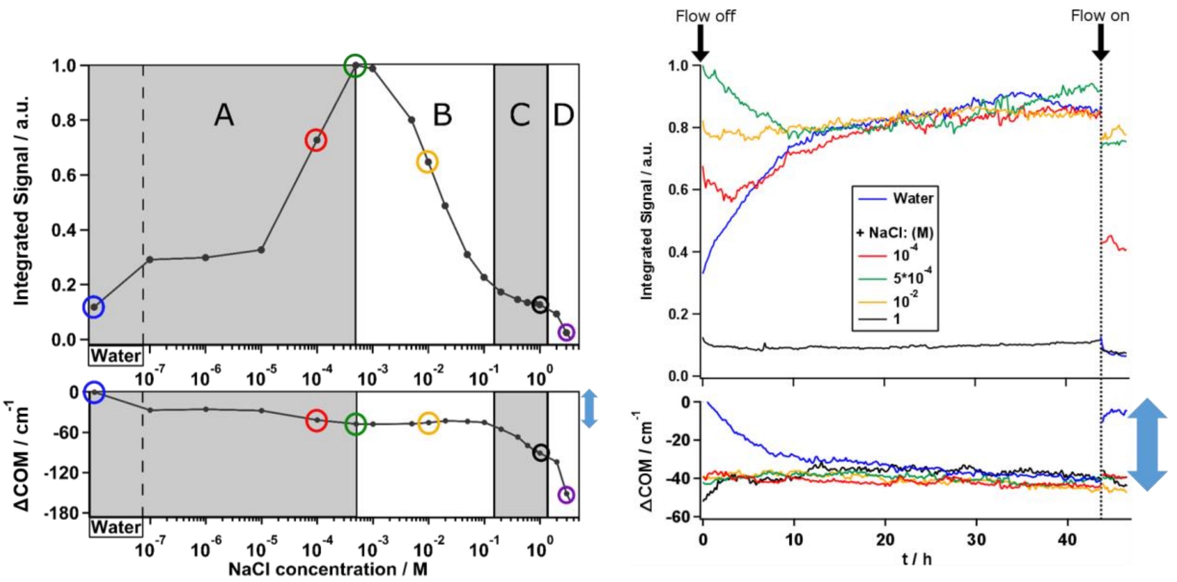

Supplementary Fig. 1: Integrated SFG signal presented in Fig. 1 and 2 in the main text, together with its center of mass frequency shift with respect to the center of mass frequency of pure water at the silica surface. The blue double arrows mark the shifts along dissolution (right) and its corresponding shift resulting from addition of NaCl to the solution (left)

Supplementary Note 2. Conversion function between SFG signal and interfacial ionic strength

In order to convert the experimental SFG data into an interfacial ionic concentration in Fig. 4, we employ the function presented in Supplementary Fig. 2 (green), that is:

$$Signal = \frac{-1}{log\left(30 * concentration\right)} \tag{S1}$$

The curvature of the required function (green) is adjusted according to the concentration-dependent SFG intensity curve (black) in Fig. 3 and is presented below in Supplementary Fig. 2.

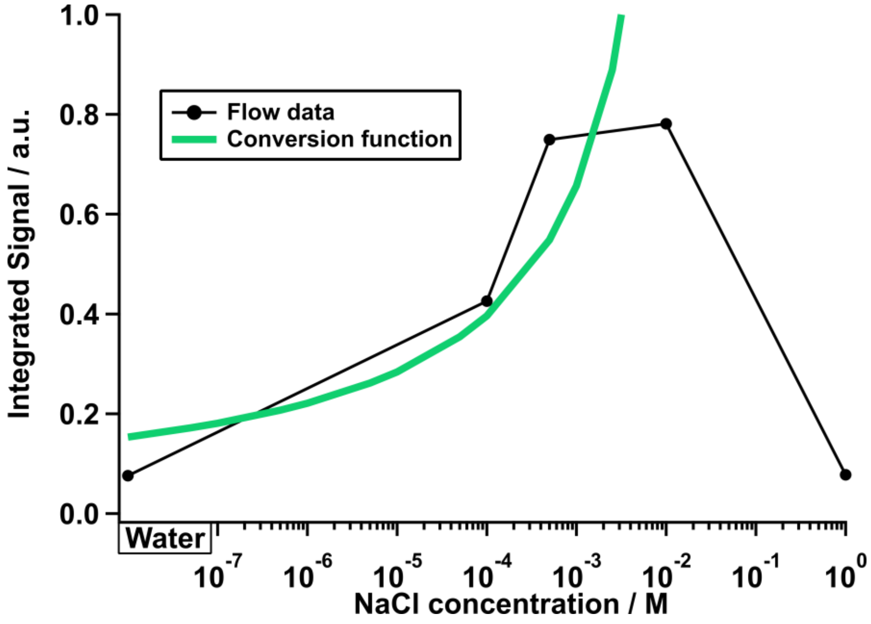

Supplementary Fig. 2: The function employed for conversion of the experimental SFG data into an interfacial concentration (depicted in Fig. 4). For comparison, the flow data presented in Fig. 3 are plotted as well.

Supplementary Note 3. Time-dependent diffusion coefficient

Supplementary Fig. 3 presents the time-dependent diffusion coefficient that gives a very similar match of the dissolution model with the experimental data presented in Fig. 4 as the invoked time-dependent dissolution rate.

$$D = 10^{-11} * e^{\left(\frac{-t}{5000}\right)} + 9.5 * 10^{-14} m^2 s^{-1} \quad \text{(S2)}$$

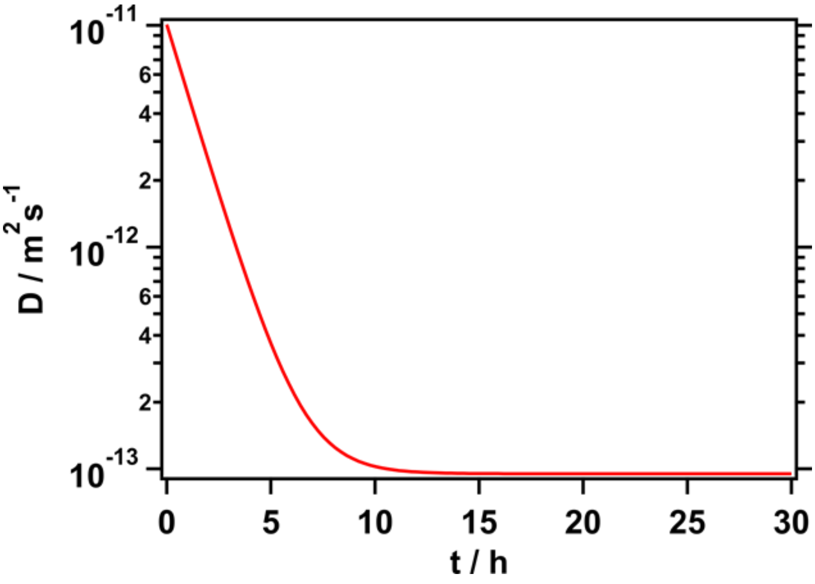

Supplementary Fig. 3: Time-dependent diffusion coefficient vs time.

Supplementary Note 4. Dissolution kinetics at pH 9

To confirm the autocatalytic mechanism of silica dissolution, mediated by hydroxide, Supplementary Fig. 4 shows the dissolution experiment for a pH 9 aqueous solution of NaOH. We observe that the kinetics speed up by over an order of magnitude compared to the experiment at neutral pH: from a tens of hours timescale to a 1-2-hour timescale under basic conditions.

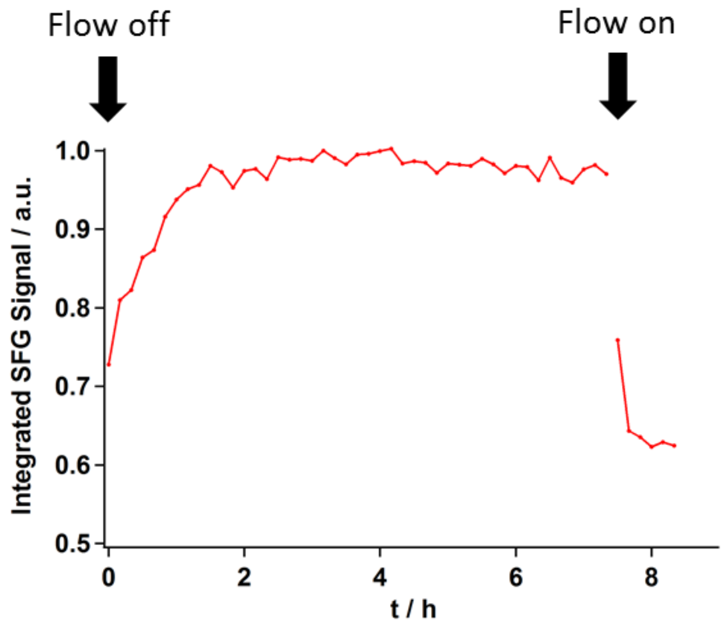

Supplementary Fig. 4: Integrated SFG signal of the silica / water interface as a function of time for a pH 9 aqueous NaOH solution

Supplementary Note 5. Time-dependent dissolution rate

For the time-dependent dissolution rate invoked in Fig. 4 (red solid line), the following functional is used:

$$k^{-1} = 8.82 * 10^{-8}\, s^{-1} \tag{S3}$$

$$k = \begin{cases} t * 1.20 * 10^{-14} + 10^{-12} mol m^{-2} s^{-1}, t < 8.5h \\ 30600 * 1.20 * 10^{-14} + 10^{-12} mol m^{-2} s^{-1}, t > 8.5h \end{cases} \tag{S4}$$

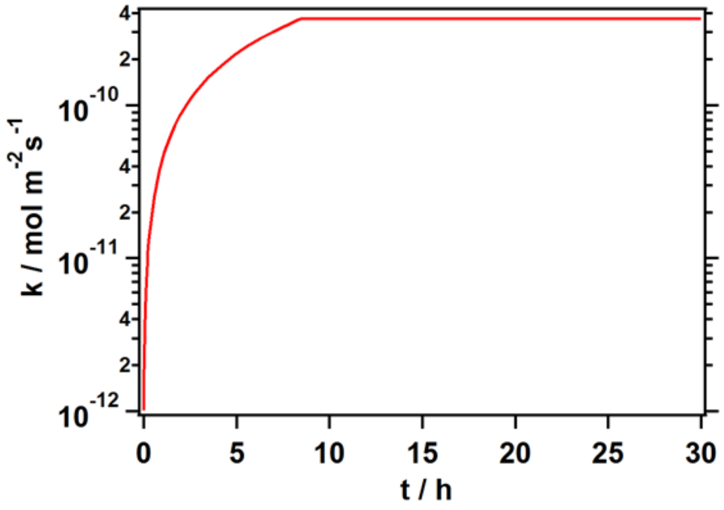

Supplementary Fig. 5: Time-dependent forward rate vs time.

Supplementary Note 6. Stability of the IR power

As introduced in the main text, part of the IR beam was separated for monitoring its stability. In Supplementary Fig. 6, a typical time trace (bottom panel) is plotted together with the time trace for the experiment with pure water presented in Fig. 2 (top panel). The plot shows IR power variation of less than 5% along the experiment.

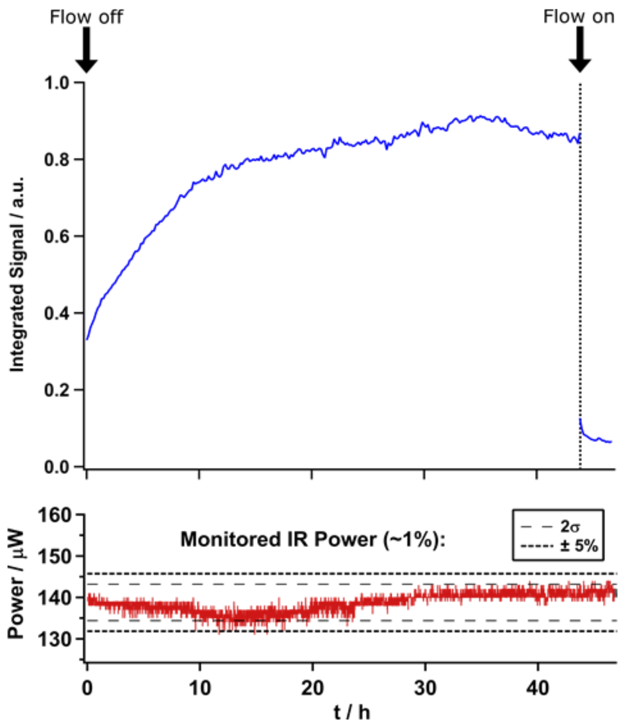

Supplementary Fig. 6: IR Power stability during experimental conditions. Top: The experiment with pure water presented in Fig. 2. Bottom: The corresponding IR Power stability along this experiment.
